# Development and Validation of an IoT Neurostimulator for the Treatment of Neurogenic Bladder

**DOI:** 10.3390/s23229284

**Published:** 2023-11-20

**Authors:** Luana Cecilia Farache Lemos Leal, Luiz Henrique Bertucci Borges, Maria Eduarda Franklin da Costa De Paula, Lilian Lira Lisboa, André Felipe Oliveira de Azevedo Dantas

**Affiliations:** 1 Graduate Program in Neuroengineering, Edmond and Lily Safra International Institute of Neuroscience, Macaíba 59280-000, RN, Brazil; luiz.borges@edu.isd.org.br (L.H.B.B.); lilian.lisboa@isd.org.br (L.L.L.); andre.dantas@isd.org.br (A.F.O.d.A.D.); 2 Orby, Co., Orby Systems Development LTDA, Natal 59000-000, RN, Brazil; eduardafranklin@orby-company.com

**Keywords:** neurogenic bladder, neuromodulation, system development

## Abstract

Neurogenic bladder is a dysfunction in the lower urinary tract due to damage to the nervous system. One of the treatments that has shown important results is transcutaneous neuromodulation. The neuromodulation equipment available on the market does not allow remote activation or offer many resources for adjusting the parameters of the generated stimulus, as most devices operate with pre-established parameters in closed packages. For this reason, customizing therapy for each individual can be difficult. Therefore, the objective was to develop and validate a neuromodulation device capable of being remotely programmed and properly monitored. Materials and methods for validating devices were used according to the Brazilian Regulatory Standard (NBR), which deals with general requirements for the basic safety and essential performance of electromedical devices. After verifying the reliability of the device, which was capable of generating a biphasic and symmetrical wave, measured by an oscilloscope, considered safe by the technical requirements, it was tested in a real application. Users reported feeling a comfortable stimulus, similar to other previously used devices, and considered the device easy to use. In this way, it was possible to demonstrate the reliability and validity of the developed device.

## 1. Introduction

The urinary bladder is a hollow organ formed by smooth muscle (detrusor), whose principal function is to store and eliminate urine in a cyclical way. In the urine storage phase (bladder filling), the detrusor musculature must relax and the urethral sphincter must contract to allow urine to accumulate inside the bladder without increasing pressure within the organ. In the emptying phase (voiding), the detrusor contracts, and the urethral sphincter relaxes to expel urine through the urethra. The functioning of the urinary bladder is coordinated, at different levels, by the central nervous system [1].

At a higher level, the cortex is responsible for the voluntary and conscious control of urination, allowing the individual to decide whether it is the appropriate time and place to urinate, respecting the volume capacity that the organ has. Sensory inputs from the bladder and sphincter provide perception of bladder capacity. These connections originate in the sacral medullary roots between levels 2 and 4 (S2 to S4), forming the pelvic plexus, which branches into the pelvic, hypogastric, and pudendal nerves. In the medulla, connections are made with neurons located in the dorsal horn [1].

The higher centers coordinate the activity of the medullary centers through the descending cephalospinal tracts. Micturition is triggered by the cortex and the connection to the brainstem, specifically by the pontine–mesencephalic substance called the Pontine Micturition Center (PMC). Urination is usually triggered by a spinal–bulbospinal reflex provided by the PMC, which receives influences, mostly inhibitory, from the cerebral cortex [1].

Any injury or change in the nerve pathways that coordinate the functioning of the urinary bladder can lead to dysfunctions that compromise the proper adjustment between the bladder filling and emptying phases [2]. This condition is called neurogenic bladder (NB) and can occur due to involvement of both the brain and spinal cord, either congenital or acquired [3]. Thus, NB can affect individuals with different neurological conditions.

NB can cause minor changes in bladder sensitivity to more serious dysfunctions such as vesico-sphincter dyssynergia capable of compromising the upper urinary tract, which comprises the ureters and kidneys [2]. Individuals with NB may have increased intravesical pressure, detrusor overactivity, incomplete bladder emptying, urinary retention, and incontinence. When untreated, the neurogenic bladder can lead to deterioration of the upper urinary tract, which can cause kidney failure, even leading the individual to death in more advanced stages and without adequate treatment [4,5].

To monitor these individuals in the context of rehabilitation, the support of a multidisciplinary team and the application of different therapeutic resources are necessary. Among the resources recommended to treat NB, the most widespread and recommended is called Clean and Intermittent Bladder Catheterization (CIBC). This consists of a bladder emptying technique that introduces a probe through the urethra into the bladder, creating a channel for the output of urine [6]. This technique is very efficient because, when performed at regular intervals, it mimics the usual cyclic functioning of bladder filling and emptying without leaving residues that could favor the proliferation of microorganisms and the installation of [7] infections.

However, the treatment of NB involves the CIBC and other techniques, such as urotherapy, anticholinergic drugs, and neuromodulation [8]. Urotherapy consists of a technique that applies behavioral adjustments to the individual’s routine, favoring better functioning of the bladder and intestine [9,10]. Anticholinergic drugs are compounds that act to promote relaxation of the detrusor musculature and prevent the so-called uninhibited contractions or detrusor overactivity that may be present in NB [11]. Also called antispasmodics, they act by blocking the muscarinic receptors present in the bladder wall [12].

So-called neuromodulation (NMD) consists of a technique that uses electrical, magnetic, or pharmacological stimuli to modulate certain areas of the nervous system [13] responsible for controlling bladder functions, with therapeutic purposes [14]. Electrotherapeutic neuromodulation is applied through devices implanted in the patient’s body or non-invasively on the individual’s skin [15]. It acts by modifying the typical tissue activity, either by inhibiting or stimulating cell activity, using electrical stimuli and can be applied to the central, peripheral, or autonomic nervous system [14,16].

One of the forms of use is the so-called transcutaneous parasacral neuromodulation, which consists of applying electrical stimuli in the parasacral region (lumbar, roots of S2 and S3). It is already a promising technique that shows good results in treating NB symptoms [17,18,19]. The detailed mechanism of action of parasacral neuromodulation is still the subject of investigation. However, it is known that the modulation of medullary reflexes and higher centers occurs through afferent pathways, from the region from S2 to S4 [20,21].

The devices available on the market are generic electrostimulators [22,23], also used for the application of *Transcutaneous Electrical Nerve Stimulation* (TENS) and *Functional Electrical Stimulation* (FES) [24]. These devices, in general, allow the modulation of the following parameters of the stimulation signal: frequency, intensity, and pulse width.

The literature presents variability in the applied current parameters. Mainly, the frequency parameters vary between 4 Hz and 20 Hz, the intensity varies between 1 mA and 100 mA, and the pulse width is between 200μs and 700μs [8]. However, most devices do not offer enough features for customizing parameters in a single device. There is also no adequate monitoring for home use, as the existing equipment does not have synchronous or asynchronous remote trigger modules.

The present work proposes the development of a device capable of remote control and programming through the internet, enabling the safe implementation of remote neuromodulation therapy for neurogenic bladder (NB). Thus, it favors telerehabilitation, in addition to accurately collecting data on stimulation voltage and current, and reducing total costs for using the resource. To date, no research on devices specifically targeting neuromodulation for NB has been identified in the literature, underscoring the importance of this work.

## 2. Materials and Methods

This experimental and descriptive study used quantitative approach strategies for the technical validation of the transcutaneous neuromodulation system for the neurogenic bladder. It was carried out within the Graduate Program in Neuroengineering at the Edmond and Lily Safra International Institute of Neuroscience (IIN-ELS) of the Santos Dumont Institute (ISD) in partnership with the Anita Garibaldi Health Education and Research Center (CEPS) of the ISD. The present work received approval from the Ethics and Research Committee (CEP) of the ISD on 23 December 2021, and was registered under the Certificate of Presentation of Ethical Appreciation (CAAE) n° 53127921.2.0000.0129.

**Figure 1:**
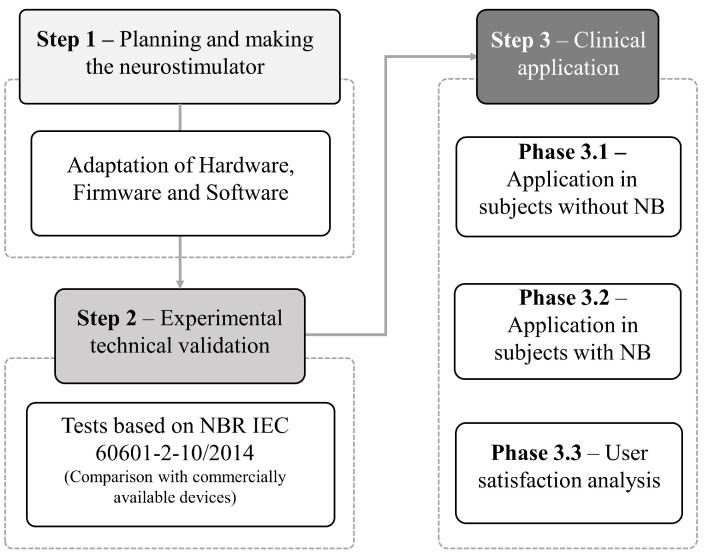
Methodological design flowchart. Source: own authorship.

The device development process took place in three main phases: planning and manufacturing of the neurostimulator, experimental technical validation, and clinical application. In the first phase, it develops *hardware*, *firmware*, and *software*, adapting De Almeida et al. [25] to the specific needs for the treatment of neurogenic bladder. In the second stage, it performs tests on the proposed hardware based on the Brazilian regulatory standard—NBR—from the Brazilian Association of Technical Standards—ANBT—which deals with general requirements for the basic safety and essential performance of electromedical devices, NBR IEC 60601-2-10/2014 [26]. The analysis results were obtained using commercially available devices as a reference. Finally, in the third stage, clinical application tests were carried out in individuals with and without NB. Then, user satisfaction questionnaires were applied. The detailed design of the methodological steps is illustrated in Figure 1.

The tests of the experimental validation stage are described below:

- Analysis for mapping and managing risks inherent to the device—Consists of a summary of the main characteristics of the device and the possible health risks associated with them (such as electric shocks and skin lesions, for example). In this phase, the system mechanisms to prevent these risks from causing iatrogenic effects to the user are identified and described.
- Current leakage measurements—In this step, the structure of the device and its connections are evaluated to avoid exposing any patient or operator to currents that are not part of the regular operation of the device.
- Functionality tests of the generated waveform—Seeks to verify the congruence between the programmed parameters of the signal and the actual parameters emitted by the device (amplitude, voltage, and others) as well as the variations in the waveform during the offer of the stimulus.
- Oscillation measurements of the generated signal—This aims to verify the modulation of the signal during changes in the stimulation parameters, as there should be no abrupt changes that harm the patient.

In the clinical application stage, tests are carried out to use the device in real applications. To carry out a reliable collection of information about the sensory perception of the stimulus it is necessary to apply neuromodulation in subjects without neurological impairment with full sensory perception capacity. This occurs because, as a result of the neurological lesion, the patient may present alterations in sensitivity (increasing or decreasing) and may even present total absence of it.

Subjects over 18 years of age, of both genders, without comorbidities or neurological diseases, participated in the clinical application stage. The sample of individuals with NB was composed of individuals with spinal cord injury followed in the neurogenic bladder care line of the CEPS of the ISD. The selection of participants with NB was based on the criteria defined in Section 2.1.

### 2.1. Inclusion Criteria

- Inclusion criteria were being at least 18 years old at the beginning of data collection and having a diagnosis of neurogenic bladder confirmed by a urologist.

### 2.2. Exclusion Criteria

- Voluntary abandonment of the research protocol;
- Diagnoses of genetic syndromes;
- Cognitive deficits that make it impossible for the user to identify and report possible changes during assessments and interventions;
- Present skin lesions or infections that prevent the fixation of self-adhesive electrodes in the parasacral region.

All subjects were invited to participate in the study voluntarily and underwent the protocol only after receiving all the necessary guidance regarding the risks and benefits associated with the device. Then, all of them signed the informed consent form according to current research ethics guidelines.

In order to analyze the patient’s perceptions of the device, the users were asked to fill out an evaluation questionnaire. At the end of the neuromodulation session, participants received a link to access the questionnaire, and answered virtually and anonymously. A questionnaire developed based on the Likert scale (LS) [27,28] was used to objectively assess the attitudes and opinions of respondents (users) in relation to their satisfaction with the proposed system. The data obtained in this step are expressed as percentages.

## 3. Results

In this section, the results obtained during the three stages of development of the proposed device presented in the Figure 1 are described.

### 3.1. System Architecture

**Figure 2:**
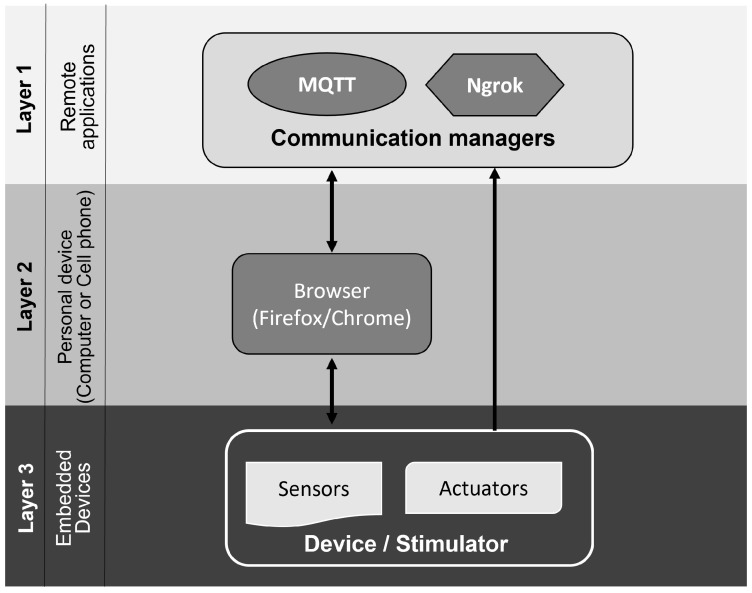
System architecture diagram. Source: own authorship.

Based on the architecture proposed by [25], we implemented some updates in the system for neuromodulation of NB. Among the innovations implemented in this work, we highlight both the modification of the hardware to allow worldwide IoT access to the device and the integration of the current sensor. Codes are implemented in an additional way to insert the routines for sending current data via MQTT (*Message Queuing Telemetry Transport*). In addition, we have developed a device management system subdivided into three layers. Figure 2 illustrates the system design.

As illustrated in Figure 2, the system consists of three main parts that communicate over the network via MQTT through the CLI tool (Comand Line Interface) Ngrok. In the upper layer there are communication management applications and intelligent services, which run remotely on a device connected to the Internet. In the secondary layer, from personal devices (computer, cell phone, etc.), page requests run, as well as the visualization of templates sent to the browser (neurostimulator operating interface). In the third and last layer, there are embedded devices, represented by the network of sensors and actuators (electrostimulators). In the case of the specific device for NB, the sensors and actuators are coupled in the same hardware, as the sensor monitors the signal emitted by the stimulator.

To implement new system components, the firmware has been modified. One of the changes is related to the readings taken by the current stimulator’s sensor. These readings are in real-time via network (service over MQTT) and for that we created a remote page to collect data from the device. This data provides useful information, such as the device’s activity status (on or off), and allows the reprogramming of parameters remotely, avoiding direct interaction with the patient.

**Figure 3:**
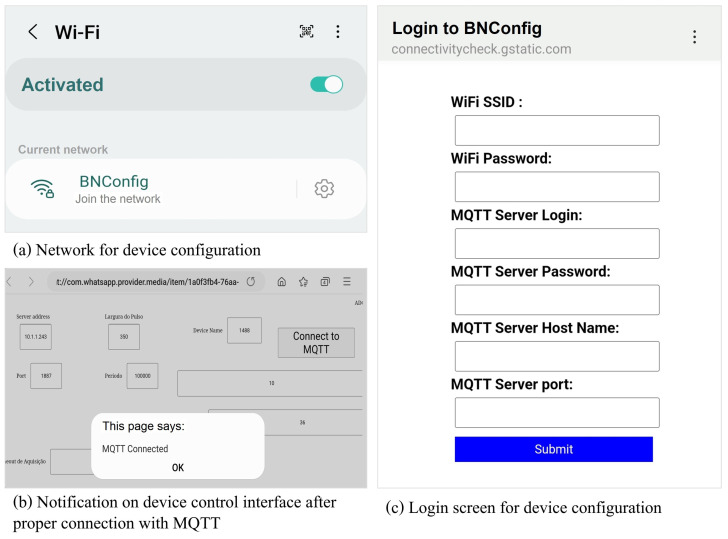
Representation of device configuration screens. Source: own authorship.

To use the device, the user (therapist or patient) must turn on the device and configure it to allow access to the WiFi network. After turning it on, the device shows a network called *NBConfig*; if the network does not appear spontaneously, the device is reset. For restarting, there is a specific button that must be pressed for 10 s in order to reconfigure the device. After connecting to the *NBConfig* network, the user is redirected to a configuration portal where the user must correctly give the name of the WiFi network and the respective access password to configure the device. Figure 3 represents the referred stages of the device configuration process.

Once connected to the internet, the device connects to the MQTT network on the mosquitto broker using Ngrok as a bridge. The infrastructure for this connection is also built on a USB stick, which can be inserted into any computer for portability, to use as a tool for managing and processing the information sent via MQTT. After connecting to the internet, the user accesses the web page that works as an interface for configuring therapy on the device. It is on this interface that the user programs the parameters of pulse duration, frequency, and current intensity. The command web page connects to the MQTT broker via websocket. The system programming routines will be in the public domain and in the GitHub repository of Santos Dumont Institute (https://github.com/isd-iin-els, accessed on 15 November 2022).

MQTT is an open protocol, which aims to promote asynchronous communication, working on top of the popular *Internet Protocol* (IP) [29]. It was designed to operate on the publish–subscribe model. Compared to other protocols, it uses as little network bandwidth resources as possible and performs better by sending only requested data. In this model, a single message is spoken and distributed to multiple users asynchronously, keeping the publisher independent of subscribers and vice versa [29].

**Figure 4:**
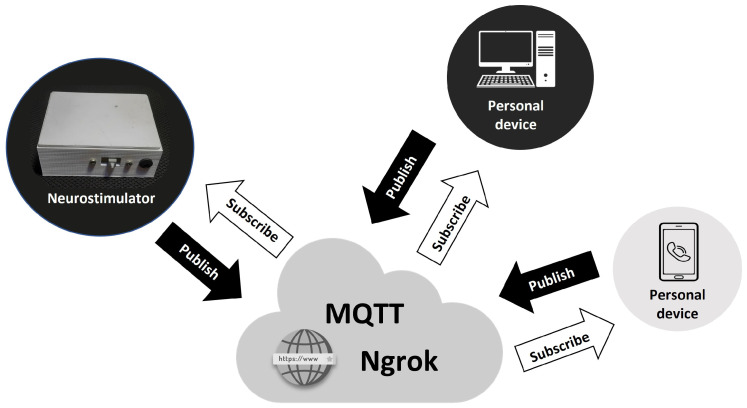
System operation to allow remote access. Source: own authorship.

In this way, to deliver the data packet with the correct stimulation parameters to the neurostimulator, the web page (on the personal device) sends a request (*publish*) via Ngrok to the MQTT Broker. Then, the neurostimulator asks the broker for a (subscribe) with the necessary data to operate. After starting the stimulation, the device sends the current sensor data (publish) through Ngrok to the MQTT Broker. Finally, this data is sent (subscribe) by the MQTT Broker via Ngrok to the tracking web page. This architecture is implemented to ensure that all system information is available remotely and can be accessed from any device (computer or cell phone). This allows the system to work regardless of the geographical distance between the device and the commanding computer or cell phone. Figure 4 illustrates this process.

**Figure 5:**
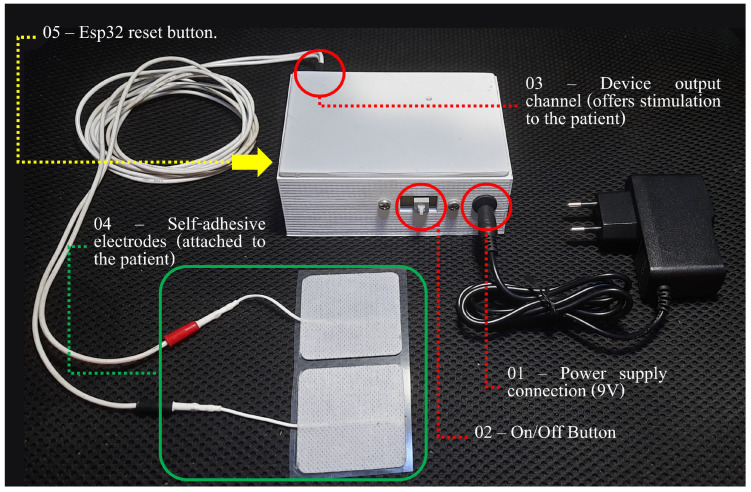
Description of the developed device. Source: own authorship.

Envisioning the possibility of using this device for telerehabilitation protocols, for patient safety, the device only has two activation buttons (one to turn on/off and the other to reconfigure the connection of the device to the WiFi network). The final version of the device is detailed in Figure 5. In order to ensure patient safety, only qualified professionals have remote access to the device. This access is via the web interface, as described above. It is through this interface that the therapist is able to define the stimulus parameters (pulse duration, frequency, and intensity), monitor whether the device is responding to voltage/current change requests in real-time (response log), view the current sensor data (log of *streaming*) and, separately, visualize the information on the devices, thus guaranteeing the effective application of the neuromodulation.

### 3.2. Hardware Construction and Adaptation

The systems currently available on the market are not specific to the NB [22,23]. They offer few resources that allow the customization and proper monitoring of the stimulation parameters [24], and do not offer remote access, which provides security and reliability for the application of home protocols. In this way, it is evident that the proposition of the present device constitutes an innovation of great relevance.

There are studies that used Arduino applications to allow the integration of several sensors simultaneously, Kciuk et al. [30]. Furthermore, for the hardware proposition, the work by De Almeida et al. [25] was used as a reference, in which an IoT Electrostimulator with Closed Loop Control was proposed. Considering that the aforementioned work is an open source and open hardware system, it is necessary to add a current sensor to monitor in real-time the amount of current applied during the stimulation session.

**Figure 6:**
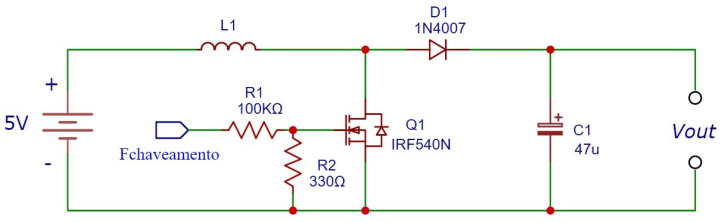
Boost schematic model. Source: De Almeida et al. (2022) [25].

In this way, the hardware is composed of four main elements. The first is a voltage booster, called *Boost*, as illustrated in Figure 6.

**Figure 7:**
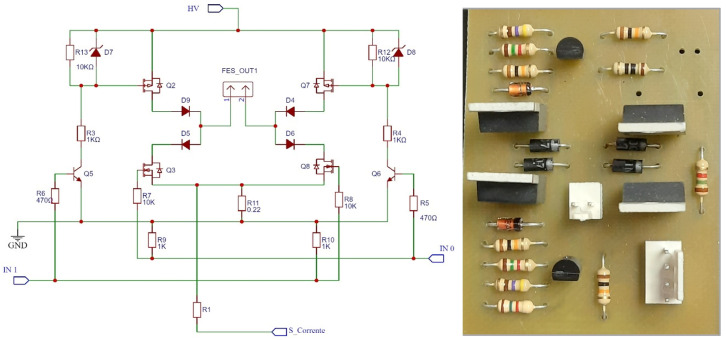
Schematic and circuit representation of the H bridge. Source: adapted from De Almeida et al. (2022) [25].

The second component is a circuit called *H Bridge*, which is responsible for generating single-phase or biphasic electrical stimuli, controlling the signal (voltage/current) and defining important parameters, such as frequency and pulse width of the signal. Its schematic is illustrated in Figure 7.

The third element is the device’s control unit, composed of the ESP32-WROOM microcontroller, produced in China by Espressif, which has a Wi-Fi module, a processor with two 32-bit Xtensa® cores and an operating frequency of up to 240 MHz.

The fourth element, the main adaptation of the circuit proposed in this work, is a current sensor. To measure the value of the stimulation current, the sensor is coupled to the output of the H.

**Figure 8:**
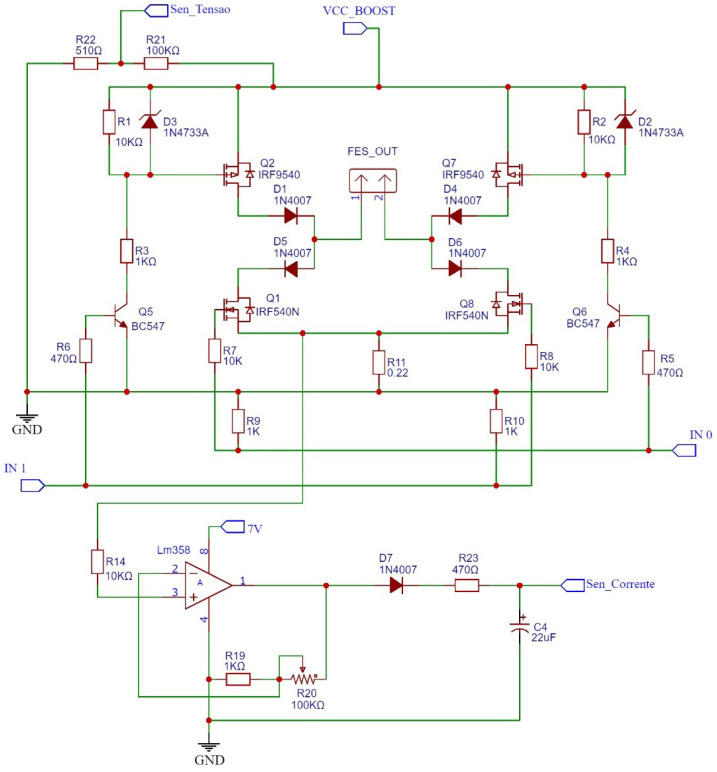
Schematic of H Bridge with current sensor. Source: adapted from De Almeida et al. (2022) [25].

The sensor circuit consists of an operational amplifier configured in non-inverting mode, which amplifies the values of the stimulation pulses to a measurable range. To filter the pulsed signal, sent by the output of the H Bridge (resistor R11), a capacitor was connected in parallel with the output of the amplifier, allowing the microcontroller to read the output current of the H Bridge. The output current of the H Bridge is the same as the one sent to the patient. The voltage is measured as the frequency or amplitude of the spikes increases, as shown in Figure 8.

### 3.3. Hardware Validation

**Figure 9:**
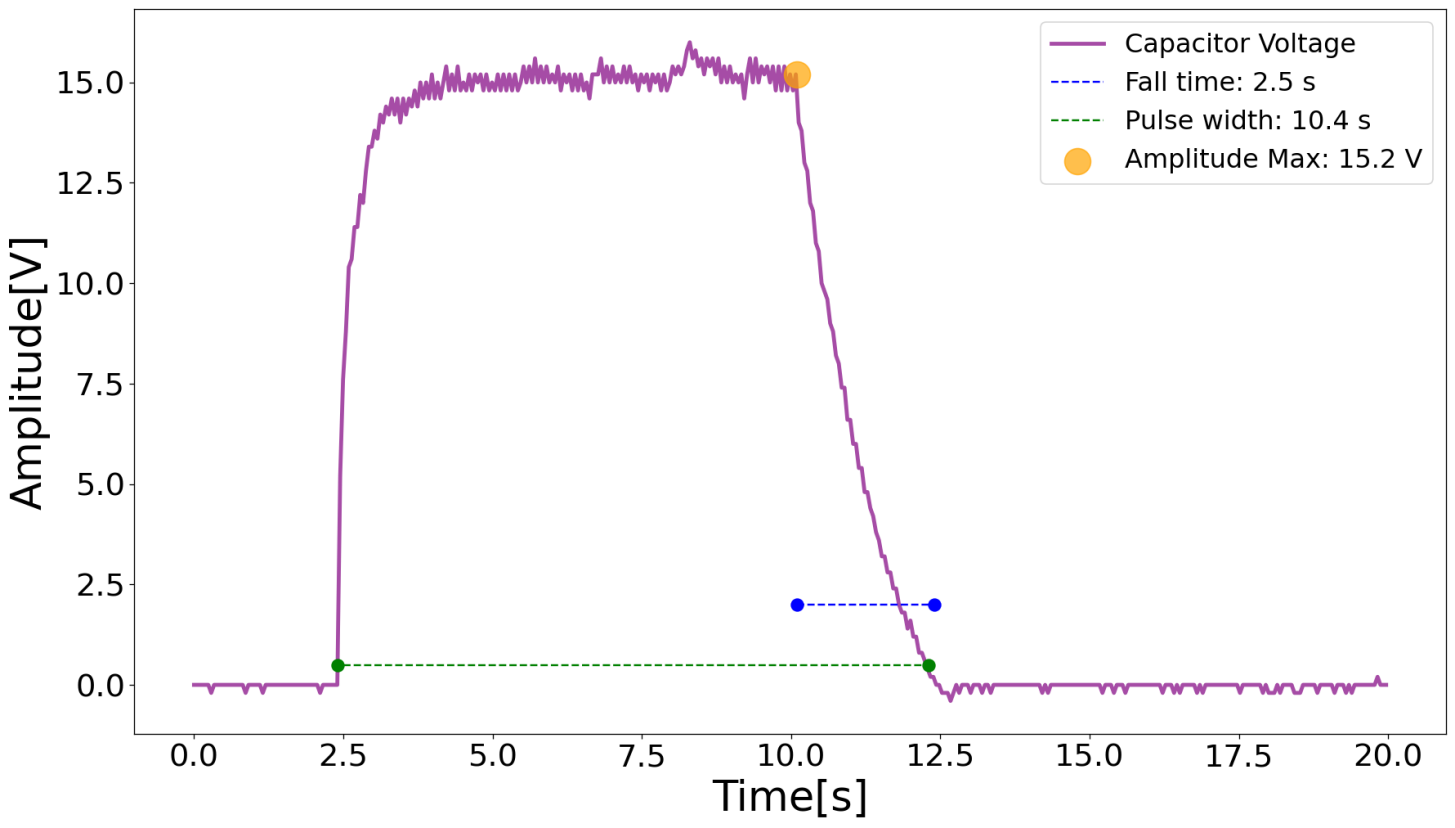
Boost capacitor discharge. Source: own authorship.

Based on the guidelines established in the “Manual for the Use of the IEC 6060 Standard” [31], in the analysis stage for mapping and managing risks inherent to the device, the following main elements were identified:

- Risk of electric shocks—Bearing in mind that the neuromodulation device is essentially an electrostimulator, which works based on electrical energy and operates by modulating the current for therapeutic purposes, the possibility of current leakage is the main risk factor to manage. To this end, in addition to proper grounding of the device’s internal circuits, an insulating interface was attached to it, made of a highly fluid polymer called *acrylonitrile butadiene styrene*, known as ABS. The box that houses the hardware circuit is made of ABS with 3D printing technology. Also, there is a risk of electric shock due to the failure of the Boost capacitor to discharge after switching off the device. For this reason, in addition to the insulating interface, tests were carried out to ensure that the capacitor discharged after the equipment was turned off, with oscilloscope measurements. The capacitor discharge is shown in Figure 9.
- Risk of injury to biological tissues—The risk of injury is related to the physiological effects of electrical current. When current passes through biological tissues, it causes changes in cell ions, triggering action potentials in nerves. To properly trigger an action potential, the stimulus needs to have an adequate amplitude and duration [32], and may provoke sensory, motor, or harmful responses, depending on the intensity of the stimulus. For this reason, it is extremely important that the device providing the stimulus is able to control and monitor the current parameters.

The circuit works with a voltage of 7 V which is supplied by connecting it to a battery or power supply. To adapt the power supply to the circuit demands, it was necessary to install a voltage regulator to internally reduce the voltage, as required by the ESP32, which needs 3.3 V. However, the source remains free for *Boost*, allowing the current control to be performed based on the information processed by the microcontroller.

For the technical validation of the equipment, performance tests were carried out on the bench to obtain a reading of the electrical stimulus generated. The device developed has only one stimulation channel, as it is for the application of neuromodulation in the neurogenic bladder. As a reference for the analysis of the obtained results, some tests were applied in a standard commercial device, and the results were compared.

**Figure 10:**
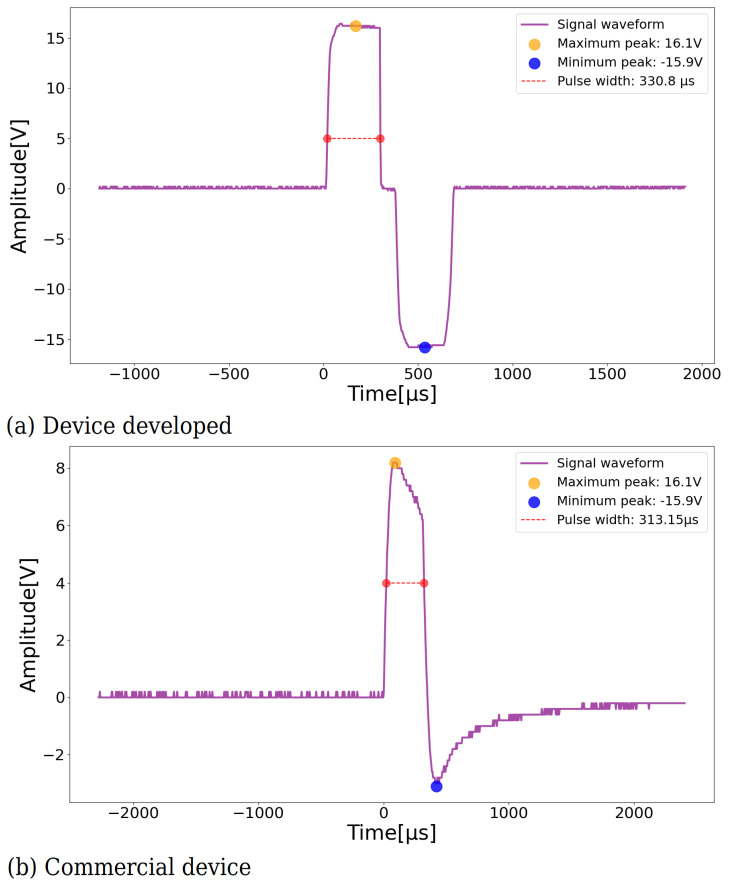
Comparison of the waveform provided by the equipment. Source: own authorship.

In the first test, the waveform of the equipment was evaluated. Figure 10a presents the results of the device developed in this work. The channel was configured to deliver biphasic waves with a pulse width of approximately 350 μs. Figure 10b represents the results obtained with a commercial device which was also programmed with a pulse width of 350 μs.

The second test evaluated the congruence between the parameters programmed in software and the parameters of the signal generated by the device through oscilloscope measurements. According to NBR IEC 60601-2-10:2014 [26] (sub-section 201.12.1.102), which deals with pulse parameters and pulse duration values, as well as pulse frequency and amplitude, it cannot present deviations greater than that ±20%.

**Figure 11:**
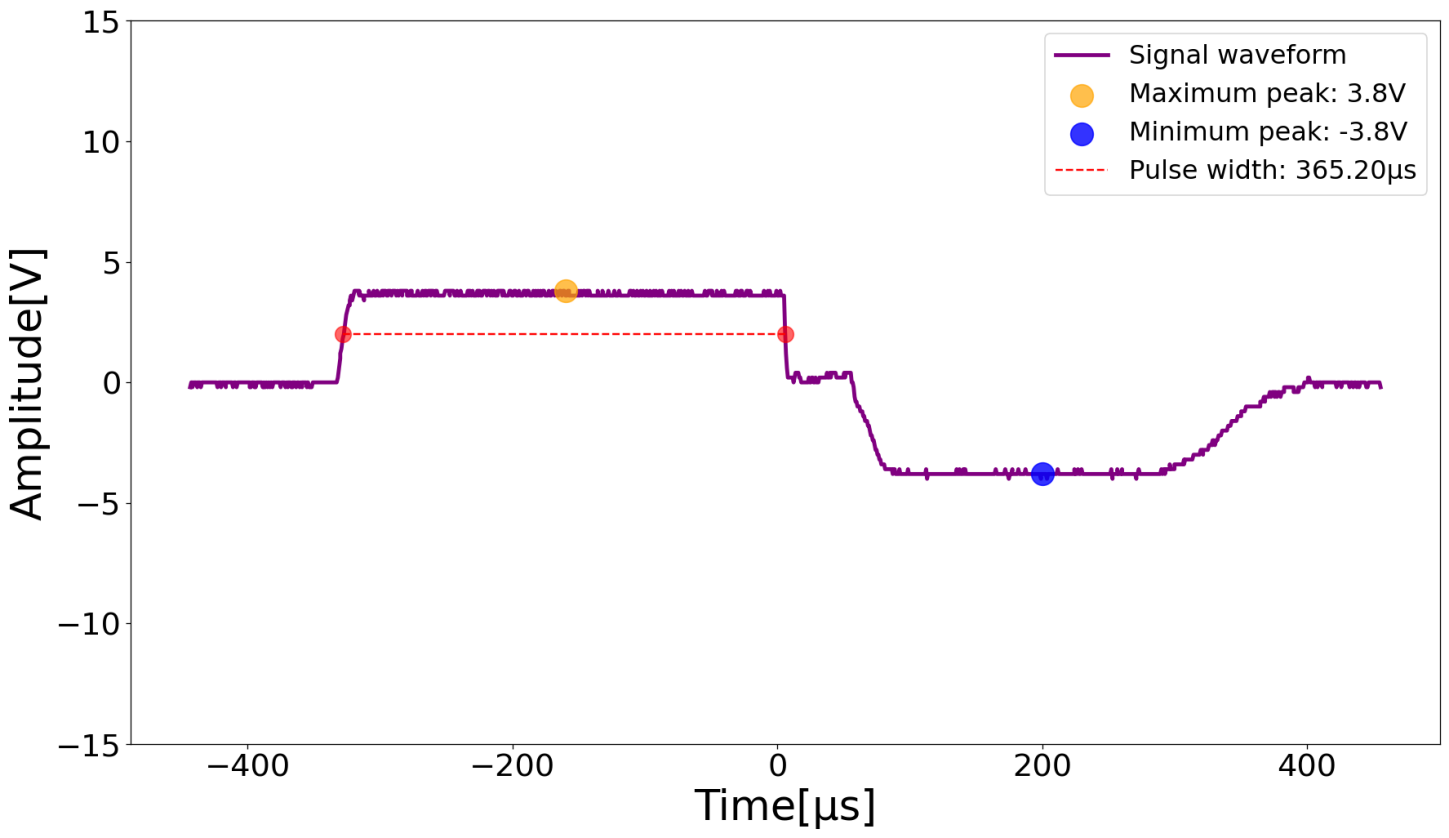
Actual signal produced. Source: own authorship.

The defined parameters were 10 Hz of frequency and 350 μs of pulse width, according to clinical protocols described in the literature [17]. In Figure 11, it is possible to observe that the parameters of the real generated signal are within the acceptable variation limit according to the regulatory standard. The time off between the positive and negative cycle of the biphasic wave may imply variations in the waveform between measurements performed with the oscilloscope.

### 3.4. Real Applications—Clinical Trials

Through a search in the literature, it was possible to identify that many studies proposed methodologies for validating electrostimulators without performing the clinical tests [33,34] stage; thus, we consider it relevant to describe the findings, even with a small sample. After signing the TFIC (Terms of Free and Informed Consent), the volunteers underwent a session of parasacral neuromodulation.

Altogether, five individuals participated, three typical subjects (one man and two women), without neurological injuries, and two individuals with spinal cord injury and neurogenic bladder (one man and one woman). The session consisted of a 30 min neuromodulation application, and the established parameters were 350 μs of pulse width and 10 Hz of frequency. The current intensity was adjusted according to the patient’s tolerance. For individuals with preserved sensitivity, the intensity was adjusted to the most intense possible, within the patient’s tolerance (the stimulus cannot be painful or considered intolerable by the patient). For individuals with altered sensitivity, commonly expressed in the form of absence of tactile sensation, the intensity was increased to the maximum, without exceeding the motor threshold; that is, the stimulus should be as intense as possible, as long as it is not enough to provoke muscle contractions.

**Figure 12:**
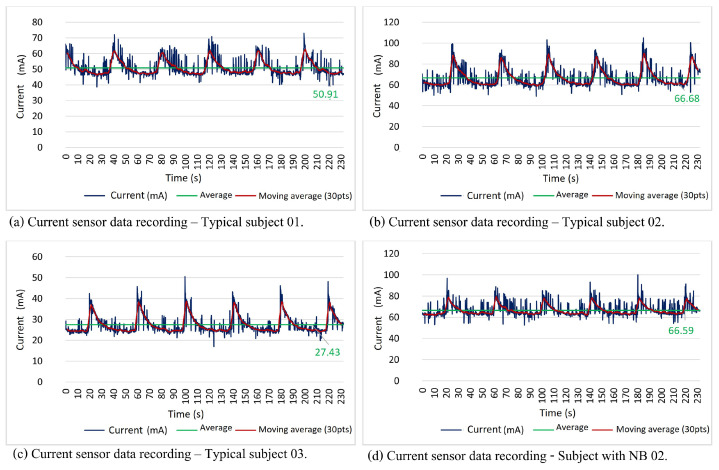
Current sensor data (current intensity over time)—subjects with full sensitivity. Source: own authorship.

In all neuromodulation application sessions, the device worked properly as it established the correct connection to WiFi and responded adequately to the data packet sent through the web interface. It was possible to monitor the current sensor data in real-time without losses or delays in the system. Figure 12 illustrates the data provided by the current intensity sensor. The therapist in charge was responsible for activating and programming the device.

The development and validation of the neurostimulator for the treatment of NB make it possible to increase the resources available for the treatment. In this way, individuals affected by neurological injuries who present with bladder dysfunctions may have easier access to technologies that enhance rehabilitation. With the help of these technologies, they will receive faster and more effective treatments with better cost-effectiveness.

Figure 12 presents the current sensor data collected after adjusting the intensity comfortable for the volunteer. It can be observed that the data provided by the current sensor demonstrate that both typical subjects and patients with NB, all with preserved sensitivity, tolerated an intensity that varied from 27.43 mA to 66.68 mA, with an average of 48.34 mA.

## 4. Discussion

The literature demonstrates that there is still no consensus on the ideal clinical parameters for the application of parasacral neuromodulation. Most devices record only pulse width and stimulus frequency values, and the intensity is adjusted only through the patient’s subjective perception. The few studies that evaluated the intensity of the current describe values ranging between 20 mA and 100 mA [8,17]. Based on these data, it was possible to observe that the first patient, with a neurogenic bladder, presented a tolerance in line with what was observed in other studies; this occurred because he had preserved sensitivity.

**Figure 13:**
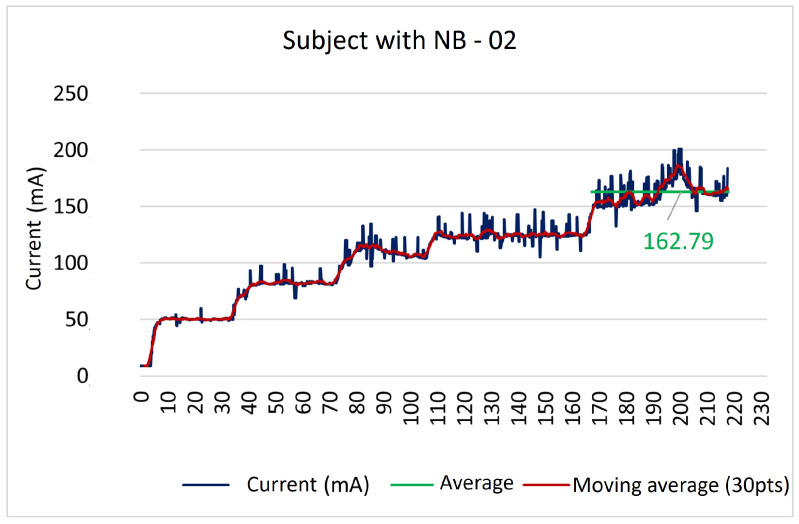
Current sensor data (current intensity over time)—subject with changing sensitivity. Source: own authorship.

However, the second patient with NB showed different behavior from the others. Her data are shown in Figure 13. This subject, in particular, presented alteration in sensitivity below the level of spinal cord injury (the level of spinal cord injury corresponds to thoracic level 10—T10, classified as complete injury by ASIA). As the patient did not have the sensitivity to perceive the stimulus, the intensity was gradually increased in an attempt to reach the motor threshold and then stimulus was reduced until the muscle contractions ceased [17].

After analyzing the current sensor reading, it was observed that the mean stimulus intensity value for this patient was around 167.79 mA. This value is above that recommended in clinical protocols that use approximately up to 100.00 mA [8,17]. This example was significant as it tested how necessary it is to insert the current sensor into the device for implementation. In addition to ensuring the correct measurement of the intensity of the current applied to the patient, it also provides information about the proper functioning of the device, which can be useful for professionals who apply neuromodulation to their patients remotely (at a distance). The absence of other studies that evaluated the exact intensity of current applications in clinical protocols prevented a better analysis of the data found in the present study. It is important to highlight that, at the end of the application, the referred patient did not present any alteration in the skin of the region where the electrode was fixed.

Some studies have proposed the creation of an electrostimulation device [33,35], but no study specifically focused on neuromodulation for neurogenic bladder was identified, evidencing the importance of this work. Most studies develop applications for functional electrostimulation [24,36]. In other works, such as Pepino [35], which also carried out device validation, a methodology similar to the present work was implemented, showing that the proposed methodological design is effective. In these cases, ANBT regulatory norms were also followed as a reference.

**Figure 14:**
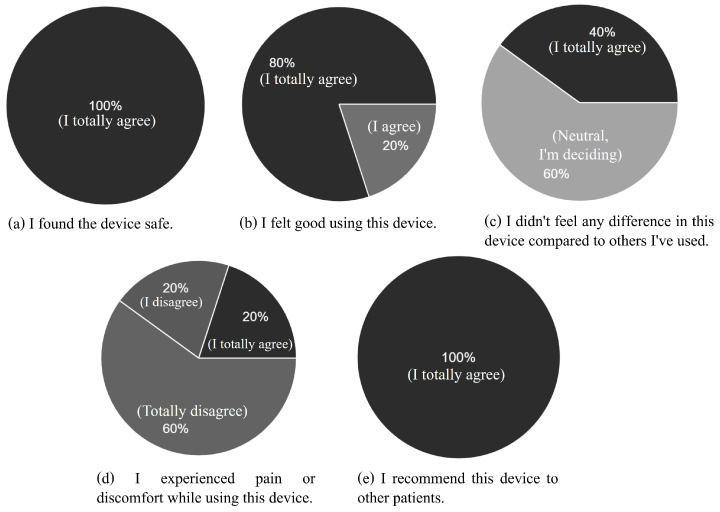
Data on user satisfaction with the device. Source: own authorship.

At the end of the parasacral neuromodulation application, the patients answered an online questionnaire regarding their perceptions of the device. The results are shown in Figure 14. Five aspects related to user satisfaction were evaluated. The aspects measured were *Neurostimulator safety—01*, *Patient’s feeling of well-being—02*, *Comparison with other devices—03*, *Stimulus discomfort—04*, and *Satisfaction with the device—05*. To quantify the evaluation of these aspects, the proposed questionnaire used the LS as a reference.

The LS is used to assess the degree of agreement or disagreement of the subjects about statements made about a given topic. It is defined as a one-dimensional scale used to assess respondents’ attitudes and opinions [37,38]. It is commonly applied in studies that assess the satisfaction of individuals in work contexts, social life, or about products and services to qualify and improve processes [39].

It is worth mentioning that, considering that most device validation studies did not perform real clinical applications [33], the conceptions and perceptions of patients are not commonly reported in the literature. The results of the present study revealed that, regarding the users’ satisfaction with the safety of the neurostimulator and the perception of well-being with the use of the device, all subjects reported being satisfied.

Regarding user satisfaction regarding the tactile perception of the stimulus offered by the device, 80% reported not feeling pain or discomfort during the application, and only 20% reported the occurrence of discomfort, but this aspect may be related to the individual characteristics of each subject. In addition to the fact that the device allows the adjustment of parameters, it allows the reduction of stimulus intensity, to make the user comfortable. In addition, all study participants reported recommending the use of the device to other patients.

## 5. Conclusions

A non-invasive IoT neurostimulator was developed and validated for the treatment of neurogenic bladder, which allows personalized adjustment of stimulation parameters, and remote and real-time monitoring of the treatment.

The hardware of the neurostimulator specific for the neurogenic bladder, which allows the personalized adjustment of the stimulation parameters, has been duly tested. From the bench measurements carried out, it was possible to verify that the device works as expected and safely.

It was also possible to develop firmware and software for the neurostimulator, which allows remote activation and monitoring of the system. Furthermore, it was possible to carry out bench and clinical tests for validation. Despite the small sample size in clinical studies, it was possible to prove the good functioning of the neurostimulator.

In general, it is expected that the present article can make a contribution and promote positive impacts for patients with NB, managers and health services, and the scientific community. In addition, it is expected that, in future work, different clinical protocols can be tested with exact control of the stimulation parameters to ensure the effectiveness of neuromodulation for neurogenic bladder, and to promote the most effective and optimized clinical improvement of patients.

## Data Availability

All system programming routines are in the public domain and are available at the Santos Dumont Institute GitHub repository (https://github.com/isd-iin-els (accessed on 22 February 2022)).

## Abbreviations

The following abbreviations are used in this manuscript:

PMC	Pontine Micturition Center
NBR	Norma Brasileira Reguladora
NB	Neurogenic Bladder
CIBC	Clean and Intermittent Bladder Catheterization
NMD	Neuromodulation
TENS	Transcutaneous Electrical Nerve Stimulation
FES	Functional Electrical Stimulation
ISD	Santos Dumont Institute
IIN-ELS	Edmond and Lily Safra International Institute of Neuroscience
CEPS	Anita Garibaldi Health Education and Research Cente
CEP	Ethics and Research Committee
CAAE	Certificate of Presentation of Ethical Appreciation
ANBT	Brazilian Association of Technical Standards
MQTT	Message Queuing Telemetry Transport
CLI	Comand Line Interface
IP	Internet Protocol
ABS	Acrylonitrile Butadiene Styrene
TFIC	Terms of Free and Informed Consent
ASIA	American Spinal Injury Association
LS	Likert scale
IoT	Internet of Things
